# Differentiating indicators of vulnerability to deliberate self-harm in forensic and non-forensic patients with schizophrenia spectrum disorders: a multinational LASSO-based analysis

**DOI:** 10.1136/bmjment-2025-302372

**Published:** 2026-09-08

**Authors:** Laura Iozzino, Donato Martella, Elena Toffol, Gabriele Torino, Elisa Caselani, Silvia Leone, Janusz Heitzman, Johannes Wancata, Marco Michael Picchioni, Giovanni de Girolamo

**Affiliations:** 1 Unit of Epidemiological Psychiatry and Digital Mental Health, IRCCS Istituto Centro San Giovanni di Dio Fatebenefratelli, Brescia, Italy; 2 Department of Statistics and Quantitative Methods, University of Milan-Bicocca, Milan, Italy; 3 University of Helsinki Faculty of Medicine, Helsinki, Finland; 4 Department of Pathophysiology and Transplantation, University of Milan, Milan, Italy; 5 Department of Forensic Psychiatry, Institute of Psychiatry and Neurology in Warsaw, Warsaw, Poland; 6 Clinical Division of Social Psychiatry, Medical University of Vienna, Vienna, Austria; 7 Department of Forensic and Neurodevelopmental Science, King’s College London Institute of Psychiatry Psychology & Neuroscience, London, UK; 8 St Magnus Hospital, Haslemere, UK

**Keywords:** Psychiatry, Schizophrenia

## Abstract

**Background:**

Deliberate self-harm (DSH) is a major public health concern among patients with schizophrenia spectrum disorders (SSDs). Although forensic patients with SSD show high rates of DSH, they remain under-represented in suicidology research, and differences in risk profiles compared with non-forensic patients are poorly understood.

**Objective:**

To compare sociodemographic, clinical, neuropsychological and criminological correlates of lifetime DSH between forensic and non-forensic patients with SSD and identify subgroup-specific vulnerability markers.

**Methods:**

Data were drawn from the multinational EU-VIORMED study across five European countries. Participants (N=392) aged 18–65 years with a Diagnostic and Statistical Manual of Mental Disorders, Fifth Edition, diagnosis of SSDs completed standardised assessments. Lifetime DSH was measured dichotomously. Separate LASSO logistic regression models identified the strongest correlates of DSH in forensic and non-forensic subsamples.

**Findings:**

Lifetime DSH prevalence was high in both groups (45.9% forensic; 39.5% non-forensic). In forensic patients, the strongest correlate was comorbid personality disorder (β=0.78), followed by functional impairment (β=0.02–0.26). In non-forensic patients, interpersonal victimisation (β=0.85) was the strongest correlate, followed by personality disorder (β=0.71), male gender (β=0.69), lifetime substance use (β=0.37) and psychopathological indicators (PANSS (Positive and Negative Syndrome Scale) items; β=−0.10–0.32). Overall, DSH in forensic patients was more strongly associated with personality pathology and functioning, whereas in non-forensic patients it was linked to trauma, clinical severity and sociodemographic factors.

**Conclusions:**

Despite similar DSH prevalence, forensic and non-forensic SSD patients showed distinct patterns of associated factors, suggesting different mechanisms of risk.

**Clinical implications:**

Suicide prevention in SSD should adopt tailored risk formulations, emphasising personality pathology and functioning in forensic settings and trauma-informed, integrated care in non-forensic services.

**WHAT IS ALREADY KNOWN ON THIS TOPIC**

- Deliberate self-harm (DSH) is common among patients with schizophrenia spectrum disorders (SSDs), and forensic patients show elevated rates of self-harm and suicide compared with non-forensic patients, but remain underrepresented in suicidology research, with their risk profiles poorly understood.

**WHAT THIS STUDY ADDS**

- Despite similar DSH prevalence in forensic (45.9%) and non-forensic (39.5%) patients with SSD, Least Absolute Shrinkage and Selection Operator (LASSO) regression identified distinct correlates: personality pathology and functional impairment in forensic patients, versus interpersonal victimisation, male gender, substance use and psychopathology in non-forensic patients.

**HOW THIS STUDY MIGHT AFFECT RESEARCH, PRACTICE OR POLICY**

- Suicide prevention in SSD should adopt tailored, context-specific approaches, emphasising personality pathology and functional autonomy in forensic settings and trauma-informed, integrated care in non-forensic services, with future research incorporating forensic-specific variables not assessed here.

Background

Deliberate self-harm (DSH), defined as a non-fatal act in which a person harms themself intentionally, with varying motives including the wish to die,^1^ represents a major public health concern, especially among those with schizophrenia spectrum disorders (SSDs). Studies indicate that the mortality rate due to suicide of patients with SSDs is approximately 5%,^2^ notably higher than the suicide rate in the general population.^3^ Therefore, the evaluation of DSH is a service priority in psychiatric care.^4^ Furthermore, self-harming behaviours, irrespective of suicidal intent, often serve as a marker of future suicide risk.^5^ Consequently, the effective identification of people at risk is imperative for the mitigation of risk by targeting clinical as well as environmental and social factors involved in suicide and self-harm processes.

A growing body of research has identified several factors associated with suicide in SSDs, including male gender, younger age, high premorbid functioning, early-stage illness, prior suicide attempts, affective symptoms and comorbid substance use or personality disorders.^6^ Furthermore, inadequate support and poor adherence to treatment have been shown to substantially elevate risk for suicidal behaviours.^7^ Several investigations have also begun to highlight the potential role of cognitive and metacognitive processes. For instance, greater cognitive insight, particularly the ability to self-reflect and recognise one’s illness, may paradoxically increase suicide risk in some patients, especially when accompanied by demoralisation and a sense of futility.^8^

Importantly, suicidal vulnerability does not necessarily coincide with overall psychopathological severity. Indeed, while more severe symptoms and poorer functioning are associated with higher odds of self-harm and suicide attempts in SSD,^9^ suicide vulnerability is largely influenced by several dimensions, including hopelessness, poor adherence to treatment, social isolation and environmental stressors.^6^ Thus, general illness severity and suicidal vulnerability represent overlapping but distinct constructs.

Within individuals with SSD, a distinct subgroup comprises those receiving care in forensic psychiatric settings and who also have a history of serious interpersonal violence. Due to their forensic status, these individuals are therefore subject to legal containment that may influence mental health outcomes, thereby representing a particularly vulnerable subgroup. Research has shown that forensic psychiatric inpatients have a higher prevalence of suicide attempts and completed suicides than non-forensic psychiatric patients.^10^ In our own multinational case-control EU-VIORMED project, 45.9% of forensic patients and 39.5% of non-forensic patients with SSD reported a previous self-harm or suicidal behaviour.^11^ These rates are consistent with estimates suggesting that up to 50% of individuals with an SSD will attempt suicide at some point during their lives.^12^ Interestingly, findings from the VIORMED-2 study revealed that psychiatric outpatients with schizophrenia and personality disorder who had a lifetime history of both interpersonal violence and self-harming behaviour exhibited more severe psychopathology than those with a history of either one alone or neither.^13^ This suggests that many variables that predict interpersonal violence (eg, substance misuse, prior violent crime and impulsivity) possibly contribute also to the risk of self-harm behaviours in individuals with SSDs.^14^ However, despite these vulnerabilities, forensic patients remain an underrepresented group within the field of suicidology, and the question of whether the underlying suicide risk profiles of forensic and non-forensic SSD patients are comparable remains unresolved. Notably, the Oxford Mental Illness and Suicide tool (OxMIS), which incorporates previous violent crime as a relevant predictor for suicide in patients with SSD and bipolar disorder, has robust predictive accuracy.^15 16^ However, the OxMIS does not explore the broader set of characteristics that may uniquely contribute to the increased vulnerability of forensic patients compared with their non-forensic counterparts. Taken together, these considerations highlight the need to disentangle the roles of diagnostic category (SSD), violent behaviour and forensic status when examining vulnerability to self-harm.

## Objectives

The present study aims to address these gaps by (1) directly comparing sociodemographic, clinical, neuropsychological and criminological characteristics that may be associated with a lifetime history of self-harm or suicidal behaviours in forensic vs non-forensic patients with SSD; and (2) assessing whether forensic status moderates these relationships. More specifically, by using a Least Absolute Shrinkage and Selection Operator (LASSO) approach to the data derived from the EU-VIORMED project, we explicitly tested for differential associations to identify both shared and subgroup-specific variables associated with self-harm behaviours, thereby offering insights that could inform risk stratification and prevention strategies tailored to specific clinical contexts.

Building on these premises, the study sets forth a series of interconnected hypotheses, as follows: (1) forensic and non-forensic patients will exhibit distinct profiles in terms of associated factors contributing to a lifetime history of DSH; (2) while certain well-established factors will be shared across both groups, the magnitude and significance of their associations with a previous intentional self-harming will differ; and (3) specific domains, such as psychosocial functioning and symptom severity, will be relevant factors associated with self-harm in forensic patients. By distinguishing between the specific factors associated with DSH in forensic and non-forensic patients with SSD, this study seeks to enhance our understanding of self-harming behaviours in forensic psychiatric populations. The findings will provide insights for clinicians, policymakers and mental health professionals in developing more effective prevention strategies tailored to the unique needs of forensic and non-forensic patients suffering from an SSD.

## Methods

### Participants

EU-VIORMED is a European multicentre observational study conducted across five countries: Austria, Germany, Italy, Poland and the UK. Participants were aged between 18 and 65 years and had a primary diagnosis of an SSD according to the Diagnostic and Statistical Manual of Mental Disorders, Fifth Edition (DSM-5) criteria, as determined by their treating clinicians. The study included two groups: a forensic patient group and a non-forensic patient group.

The forensic group consisted of individuals with an established history of serious interpersonal violence, from various forensic psychiatric facilities in each participating country (see online supplemental material S1). Serious interpersonal violence was defined as having committed a homicide, attempted homicide or other forms of assault resulting in severe physical harm to another person.

10.1136/bmjment-2025-302372.supp1 Supplementary data

The non-forensic group comprised patients matched by age and gender but no history of such violence. They were recruited from psychiatric residential facilities (inpatients) or general psychiatric services (outpatients).

The following exclusion criteria were applied: (1) confirmed intellectual disability; (2) history of traumatic brain injury or organic brain disorder; (3) insufficient fluency in the national language; and (4) scheduled discharge from psychiatric care within the following month.

### Recruitment

At each site, treating clinicians invited potentially eligible participants to take part in the study. Participants received written information detailing the study’s purpose and procedures and were given the opportunity to ask questions.

The initial target was to recruit 200 forensic and 200 gender-matched and age-matched non-forensic patients. However, the global COVID-19 pandemic led to temporary halts in recruitment across all countries.

The study received ethical approval from the Research Ethics Committee (EC) of the coordinating centre (IRCCS Centro San Giovanni di Dio Fatebenefratelli, Brescia, Italy: no. 74-2018), and from the appropriate ethics committees for each of the other countries (see the Ethics section at the end of the paper). All participants provided written informed consent to participate. All procedures contributing to this study comply with the Helsinki Declaration of 1975, as revised in 2013.

For more details about the EU-VIORMED study, see de Girolamo *et al*.^11^

### Sociodemographic, clinical, functional and violence assessment

All participants were assessed by centrally trained research assistants employed by the study. Sociodemographic, core clinical, criminological and violence risk data were collected using a study-specific Patient Information Form (PIF), an Index Violence Sheet (IVS), and a Risk Factors Questionnaire (RFQ). These were administered through patient interview cross-referenced with medical and criminal records and reviewed by clinicians.

As part of the EU-VIORMED project, the presence of self-harming behaviour and/or suicide attempts over the patient’s lifetime was assessed using a specific binary question (yes/no) included in the PIF (item 23, see online supplemental material S2). In the context of this study, a lifetime history of DSH was defined as: ‘a non-fatal act in which a person harms himself or herself intentionally, with varying motives including the wish to die’,^1^ and corresponds to the International Classification of Diseases, Tenth Revision (ICD-10) definition of intentional self-harm (X60-X84).

10.1136/bmjment-2025-302372.supp2 Supplementary data

DSM-5 diagnoses were based on the treating clinicians’ evaluations in the medical records. Current psychotic symptoms were evaluated using the original standard model for the Positive and Negative Syndrome Scale (PANSS),^17^ through a semistructured interview and clinical observation. All research assistants received centralised PANSS training in 2018 from the *PANSS Institute* and were certified as qualified raters.

The WHO Disability Assessment Schedule 2.0 (WHODAS 2.0)^18^ was used to measure everyday functioning across six domains.

Care needs were assessed using the Camberwell Assessment of Need (CAN),^19^ an interview-based tool designed for both patients (CAN-P) and clinical staff (CAN-S). Forensic patients’ needs were assessed using the Camberwell Assessment of Needs – Forensic Version (CANFOR),^20^ adapted from the original CAN that includes three additional items specifically relevant to forensic populations.^20^ In this study, we considered only the 22 items shared between the CAN and the CANFOR.

### Cognitive assessment

Cognitive functioning was evaluated using the Brief Assessment of Cognition in Schizophrenia (BACS),^21^ which measures six cognitive domains.

Patients’ decision-making capacity related to their current treatment was assessed using the MacArthur Competence Assessment Tool for Treatment (MacCAT-T).^22^

All assessment tools were administered using the respective officially translated and validated versions.

### Statistical analyses

Continuous variables are expressed as means with SD, while categorical variables are presented as counts and percentages. Group differences between forensic and non-forensic patients were evaluated using t-tests or the Mann-Whitney test for continuous variables, depending on the normality of the distribution, which was assessed using the Shapiro-Wilk test and graphical inspection. For categorical variables, χ^2^ tests or Fisher’s exact test were employed as appropriate. To avoid redundancy with item-level variables, the final design matrix included 88 candidate predictors after excluding aggregate scale scores. In the forensic subsample, 101 participants reported a history of DSH while 68 participants reported a history of DSH in the non-forensic subsample. Given the relatively high dimensionality of the predictor set in relation to the number of outcome events, we used penalised regression (LASSO) to reduce the risk of overfitting and to identify the most relevant predictors. For each group, the predictors were standardised and a design matrix constructed. A 10-fold cross-validation was employed to identify the optimal penalisation parameter that minimised binomial deviance. Final LASSO models were then fitted using the *glmnet* function with an alpha of 1 and the selected minimum lambda. Only the variables with non-zero coefficients were retained as relevant predictors. The resulting β coefficients show the direction and relative strength of each variable’s association with the outcome in the penalised model rather than providing unbiased effect size estimates like standard regression. Larger absolute values indicate greater importance in predicting DSH. In addition, sensitivity analysis excluding CAN-CANFOR item 10 (self-harm behaviours and suicidal ideation) was presented in online supplemental material S3.

10.1136/bmjment-2025-302372.supp3 Supplementary data

To formally evaluate whether the associations between the identified predictors and lifetime self-harm varied between forensic and non-forensic patients, we conducted additional regression models that included interaction terms with group status. Given the relatively high prevalence of the outcome, we employed modified Poisson regression models with robust standard errors to estimate risk ratios (RRs) and corresponding 95% CIs. First, we fitted separate models for each predictor, incorporating the interaction term between the predictor and subject type (forensic vs non-forensic) to explore possible moderation effects. Next, we estimated a multivariable model that included all selected predictors along with their interaction terms with subject type. This final model aimed to assess whether the associations differed between the two groups after accounting for mutual adjustments. All analyses were performed using R (V.4.4.0), with statistical significance set at p<0.05.

## Findings

### Sociodemographic and clinical characteristics

Out of 575 patients who initially expressed interest in participating, 175 declined to enrol, 99 forensic (30.9%) and 76 non-forensic patients (30.0%). Refusal rates in both groups varied significantly across the five participating countries (p=0.002 for forensic; p<0.001 for non-forensic). Specifically, refusal rates among forensic patients were lower in Poland than in the other countries, while refusal rates among non-forensic patients were higher in Germany and Poland (see online supplemental Table 2S in de Girolamo et al.11). Due to ethical constraints, no data were collected on those who refused to participate.

The final sample included 392 patients with a primary diagnosis of an SSD: 220 forensic patients and 172 non-forensic patients. The two groups did not differ significantly in age (p=0.223). Most participants were male (n=332; 84.7%). No significant differences were found between forensic and non-forensic patients in terms of marital or occupational status or social support (see table 1). However, forensic patients had significantly lower educational attainment compared with non-forensic patients (p≤0.001) and were more likely to have children (p=0.015).

**Table 1:** Sociodemographic and clinical characteristics of the sample (N=392)

Variable	Forensic (n=220)	Non-forensic (n=172)	P value
Age (years)	39.2 (11.2)	37.6 (11.6)	0.223
Gender			
Male	194 (88.2%)	138 (80.2%)	0.103
Female	26 (11.8%)	34 (19.8%)	
Self-harm behaviours			
Yes	101 (45.9%)	68 (39.5%)	0.348
No	119 (54.1%)	104 (60.5%)	
Disease duration (years)	13.3 (9.6)	13.9 (10.6)	0.659
Education years	11.5 (3.3)	12.9 (3.5)	**<0.001**
Diagnosis			
Schizophrenia	174 (79.1%)	127 (73.8%)	**0.004**
Schizoaffective disorder	21 (9.5%)	39 (22.7%)	
Delusional disorder	12 (5.5%)	1 (0.6%)	
Brief psychotic disorder	1 (0.4%)	1 (0.6%)	
Schizophreniform disorder	5 (2.3%)	1 (0.6%)	
Drug-induced psychosis	7 (3.2%)	3 (1.7%)	
Children			
Yes	56 (25.5%)	23 (13.4%)	**0.015**
No	164 (74.5%)	149 (86.6%)	
Marital status			
Married	10 (4.5%)	14 (8.1%)	0.348
Single	182 (82.7%)	140 (81.4%)	
Widowed, divorced	28 (12.7%)	18 (10.5%)	
Occupation			
Yes	188 (85.5%)	148 (86.0%)	0.983
No	32 (14.5%)	24 (14.0%)	
Social support			
Yes	182 (82.7%)	154 (89.5%)	0.154
No	38 (17.3%)	18 (10.5%)	
Personal contacts			
Yes	102 (46.4%)	155 (90.1%)	**<0.001**
No	118 (53.6%)	17 (9.9%)	
Witnessed violence			
Yes	71 (32.3%)	45 (26.2%)	0.348
No	149 (67.7%)	127 (73.8%)	
Victim violence			
Yes	79 (35.9%)	52 (30.2%)	0.348
No	141 (64.1%)	120 (69.8%9	
Beaten, kicked or punched			
Yes	152 (69.1%)	93 (54.1%)	**0.012**
No	68 (30.9%)	79 (45.9%)	
Lifetime substance use			
Yes	170 (77.3%)	126 (73.3%)	0.463
No	50 (22.7%)	46/26.7%)	
Comorbidity with personality disorders			
Yes	65 (29.5%)	13 (7.6%)	**<0.001**
No	155 (70.5%)	159 (92.4%)	
Type of PD			
Paranoid	2 (0.9%)	1 (0.6%)	**<0.001**
Schizoid	1 (0.5%)	0 (0.0%)	
Schizotypal	6 (2.7%)	2 (1.2%)	
Borderline	13 (5.9%)	2 (1.2%)	
Narcissistic	4 (1.8%)	0 (0.0%)	
Histrionic	0 (0.0%)	1 (0.6%)	
Antisocial	28 (12.7%)	0 (0.0%)	
Avoidant	0 (0.0%)	0 (0.0%)	
Dependent	1 (0.5%)	2 (1.2%)	
Obsessive-compulsive	2 (0.9%)	1 (0.6%)	
Missing	8 (3.6%)	4 (2.3%)	
PANSS total score	66.4 (22.0)	67.0 (17.7)	0.782
BACS total score	191.4 (46.4)	207.7 (50.5)	**0.002**
MacArthur total score	14.2 (4.6)	15.6 (3.9)	**0.002**
WHODAS total score	8.1 (8.3)	13.3 (8.4)	**<0.001**
CAN/CANFOR total score	9.4 (5.3)	8.1 (4.3)	**0.018**

P-value in bold are <0.05

BACS, Brief Assessment of Cognition in Schizophrenia; CAN, Camberwell Assessment of Need; CANFOR, Camberwell Assessment of Needs – Forensic Version; PANSS, Positive and Negative Syndrome Scale; WHODAS, World Health Organization Disability Assessment Schedule.

Clinical characteristics are presented in table 1. The most frequent primary diagnoses across both groups were schizophrenia (76.4%). The groups differed significantly in the mean age at first contact with psychiatric services. Forensic patients were significantly more likely to have a comorbid personality disorder diagnosed than non-forensic patients (p<0.001), particularly antisocial personality disorder (12.7% vs 0%). No significant group differences emerged in lifetime alcohol or substance use problems (p=0.463). Additionally, there were no differences between the groups regarding self-reported exposure to, or victimisation from, familial physical or sexual violence (p=0.348). However, forensic patients were more likely to report being victims of violence outside the family setting (p=0.012).

There were no significant differences in lifetime DSH between the two groups (p=0.348).

In terms of current psychopathology, there were no significant differences between forensic and non-forensic patients in symptom severity in the PANSS total score (p=0.782). However, forensic patients had better social functioning on the WHODAS, with lower levels of disability (mean score: 8.1, SD=8.3 for forensic vs 13.3, SD=8.4 for non-forensic patients; p<0.001). In contrast, cognitive performance, as measured by the BACS, was significantly lower in forensic than in non-forensic patients (p=0.002).

### Factors associated with self-harm behaviours in forensic and non-forensic psychiatric patients

**Figure 1:**
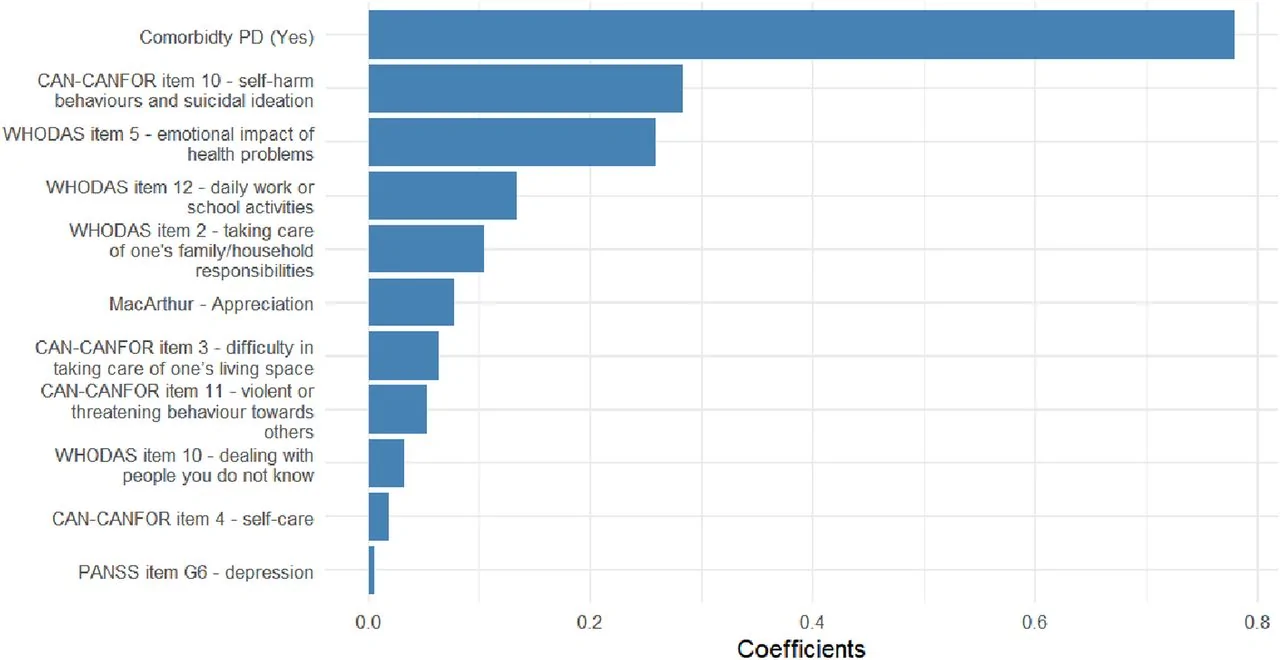
LASSO regression analysis of factors associated with self-harm behaviours in forensic patients (n=220). CAN, Camberwell Assessment of Need; CANFOR, Camberwell Assessment of Needs – Forensic Version; LASSO, Least Absolute Shrinkage and Selection Operator; PANSS, Positive and Negative Syndrome Scale; PD, Personality Disorders; WHODAS, World Health Organization Disability Assessment Schedule.

In the forensic patient sample, the LASSO regression analysis identified several factors associated with lifetime DSH. The strongest positive associated variable was the presence of a comorbid personality disorder (β=0.78). Functional assessment measures also contributed significantly, with CAN-CANFOR item-10 (β=0.28, self-harming behaviours and suicidal ideation) and WHODAS item-5 (β=0.26, emotional impact of health problems) identified as important factors. Additional functional impairment indicators linked to self-harm and suicidal behaviour included WHODAS item-12 (β=0.13, daily work or school activities) and WHODAS item-2 (β=0.10, taking care of one’s family/household responsibilities). Clinical measures had a modest impact on the results, with the MacArthur Appreciation Scale (β=0.08), CAN-CANFOR item-3 (β=0.06, difficulty in taking care of one’s living space) and CAN-CANFOR item-11 (β=0.05, violent or threatening behaviour towards others) showing smaller, yet still meaningful, associations. Minimal contributions were detected for WHODAS item-10 (β=0.03, dealing with people you do not know), CAN-CANFOR item-4 (β=0.02, self-care) and PANSS item-G6 (β=0.01, depression) (figure 1).

**Figure 2:**
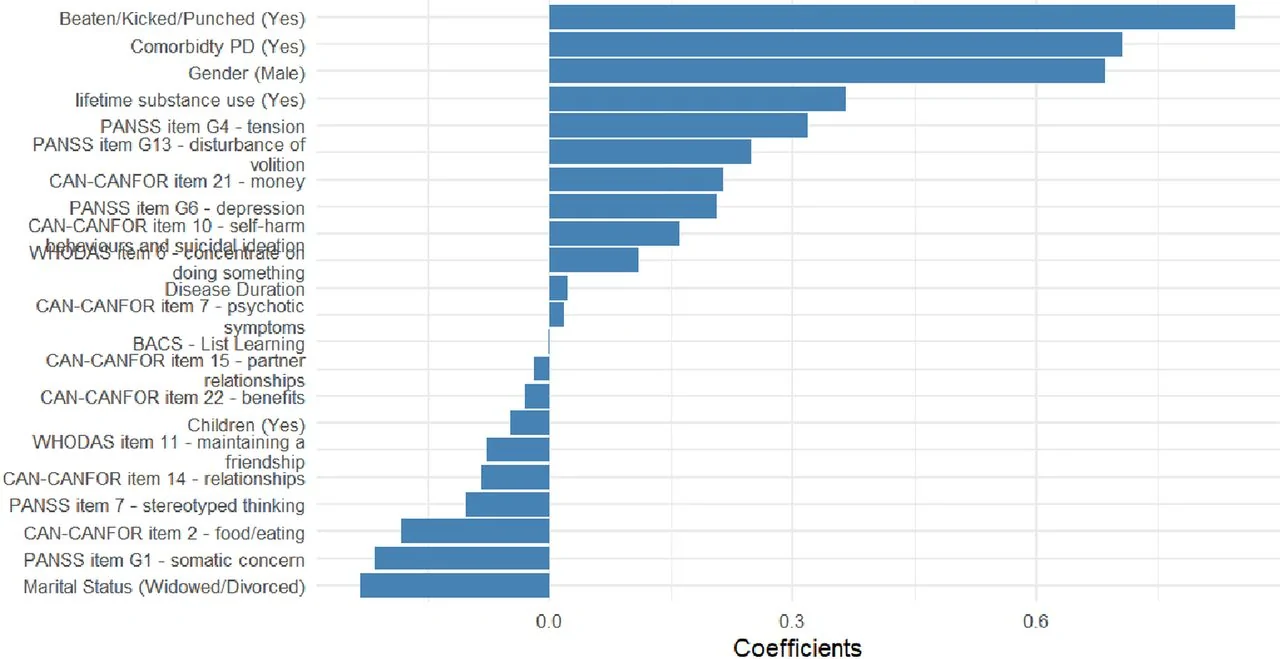
LASSO regression analysis of factors associated with self-harm behaviours in non-forensic patients (N=172). CAN, Camberwell Assessment of Need; CANFOR, Camberwell Assessment of Needs – Forensic Version; LASSO, Least Absolute Shrinkage and Selection Operator; PANSS, Positive and Negative Syndrome Scale; PD, Personality Disorders; WHODAS, World Health Organization Disability Assessment Schedule.

The non-forensic patient sample yielded a broader range of items associated with a lifetime history of DSH. The strongest association was found with a history of being beaten, kicked or punched (β=0.85), followed closely by the presence of a comorbid personality disorder (β=0.71). Male gender (β=0.69) was an associated variable, along with lifetime substance use (β=0.37). In terms of current symptoms, several measures were associated with DSH including PANSS item G4 (β=0.32, tension), PANSS item G13 (β=0.25, disturbance of volition) and CAN-CANFOR item 21 (β=0.21, money). Additional factors associated with DSH behaviours included PANSS item G6 (β=0.20, depression), CAN-CANFOR item 10 (β=0.16, self-harming behaviours and suicidal ideation) and WHODAS item 6 (β=0.11, concentrate on doing something). Several other variables contributed less significantly, such as disease duration (β=0.02), various CAN-CANFOR items, BACS list learning (β=−0.01), having children (β=−0.05), WHODAS item 11 (β=−0.08, maintaining a friendship), PANSS item N7 (β=−0.10, stereotyped thinking) and marital status (β=−0.23, specifically being widowed or divorced) (figure 2).

Overall, differences were observed between forensic and non-forensic patient groups. In forensic patients, having a comorbid personality disorder was the strongest item associated with DSH, while non-forensic patients displayed a more complicated risk profile that included factors such as a history of trauma, demographic variables (eg, male sex) and substance use. In terms of functional impairment, WHODAS and CAN-CANFOR items were notably significant for forensic patients, while current symptom severity, as measured by the PANSS, was more common among non-forensic patients. This suggests that the underlying mechanisms for suicide and self-harm risk may differ between these two populations. Specifically, trauma exposure was a particularly strong factor associated with DSH for non-forensic patients, while forensic patients were more influenced by personality pathology, functional capacity and needs.

### Interaction analyses

Interaction analyses (see table 2) were performed to assess whether the relationships between the selected predictors (the first five) and lifetime self-harm varied between forensic and non-forensic patients. In the univariate models, only lifetime substance use demonstrated a statistically significant interaction with the type of subject (RR=0.82, 95% CI 0.70 to 0.96, p=0.015). This suggests that the link between substance use and self-harm is weaker in forensic patients compared with non-forensic patients.

**Table 2:** Interaction analyses between forensic and non-forensic patients for the predictors examined

Interaction term	Univariate		Multivariable	
	Estimate, 95% CI	P value	Estimate, 95% CI	P value
Comorbidity PD	0.97 (0.81 to 1.16)	0.708	1.12 (0.92 to 1.36)	0.257
CAN–CANFOR 10	1.01 (0.90 to 1.13)	0.857	0.95 (0.84 to 1.09)	0.523
WHODAS S5	1.04 (0.99 to 1.09)	0.086	1.06 (0.98 to 1.12)	0.056
WHODAS S12	1.03 (0.97 to 1.10)	0.277	1.05 (0.98 to 1.13)	0.169
WHODAS S2	1.04 (0.98 to 1.11)	0.152	1.03 (0.96 to 1.11)	0.368
Beaten, kicked or punched	0.93 (0.81 to 1.08)	0.381	0.88 (0.79 to 1.01)	0.055
Gender	0.94 (0.79 to 1.14)	0.552	0.86 (0.73 to 1.01)	0.073
Lifetime substance use	0.82 (0.70 to 0.96)	0.015	0.85 (0.73 to 0.98)	0.0438
PANSS G4	0.95 (0.90 to 1.01)	0.087	0.93 (0.88 to 0.99)	0.013

CAN, Camberwell Assessment of Need; CANFOR, Camberwell Assessment of Needs – Forensic Version; PANSS, Positive and Negative Syndrome Scale; WHODAS, World Health Organization Disability Assessment Schedule.

In the multivariable model, which included all predictors simultaneously, lifetime substance use (RR=0.85, 95% CI 0.73 to 0.98, p=0.044) and PANSS G4 (tension) (RR=0.93, 95% CI 0.88 to 0.99, p=0.013) exhibited statistically significant interaction effects. These results indicate that the relationship between these variables and lifetime self-harm differed between forensic and non-forensic patients.

For the other predictors, including personality disorder comorbidity and measures of functional impairment, no statistically significant interaction effects were found, suggesting that the associations across both groups were generally comparable.

## Discussion

This study aimed to identify and compare factors associated with lifetime DSH between forensic and non-forensic patients with SSD. Our findings underscore both shared and distinct risk profiles across these two populations, offering some critical insights for suicide risk stratification and prevention strategies tailored to different clinical contexts.

Consistent with existing literature, the overall prevalence of lifetime DSH in our sample was high, affecting 45.9% of forensic and 39.5% of non-forensic patients, with no statistically significant difference between groups. This finding aligns with prior multinational and national studies^11^ and highlights the importance of routine systematic suicide risk assessment in all patients with SSD, irrespective of clinical context.

The LASSO regression analyses revealed a narrower and more clinically focused set of factors associated with lifetime DSH in forensic patients, as opposed to a more complicated and broader risk profile among non-forensic patients with SSD. In the forensic group, the presence of a comorbid personality disorder, particularly antisocial personality disorder, emerged as the strongest variable associated with DSH. This finding is consistent with previous research linking antisocial traits to heightened impulsivity, poor affect regulation and increased risk for both interpersonal violence and self-harm behaviour.^23^ Specific antisocial traits, including Machiavellianism, psychopathy, sadism and trait aggression, have been positively associated with non-suicidal self-injury, suggesting a complicated interplay between these traits and self-harming and suicidal behaviours.^24^ Among male prisoners, additional predictors of suicidal behaviour include early-life trauma, a history of violent offences, institutional misconduct, low levels of social support and impulsivity.^25^ Taken together, these overlapping vulnerabilities suggest that developmental processes rooted in both trauma and personality factors may underpin at least some suicidal behaviour in forensic populations.

Functional impairment, particularly in social participation and interpersonal relationships, as captured by specific WHODAS and CANFOR items, also featured prominently. These results suggest that, in forensic populations, disruptions in current functional abilities and interpersonal domains may exacerbate feelings of isolation or hopelessness,^26^ increasing DSH risk even in the absence of severe psychopathological symptoms.

Conversely, the suicide risk profile in non-forensic patients appeared more multifaceted. Beyond the presence of a personality disorder, the strongest predictors were a history of interpersonal victimisation, particularly experiences of being beaten, kicked or punched. These findings underscore the central role of trauma exposure in shaping suicide risk in this population, aligning with a growing body of evidence that links physical and emotional abuse to suicidal ideation and behaviour.^10^ Childhood maltreatment, and in particular sexual abuse, have been identified as strong risk factors for self-harm, while other adverse early life experiences may exert indirect effects on suicidal risk.^27^

Furthermore, sociodemographic and behavioural variables such as male gender, substance use and marital status (being widowed or divorced) emerged as relevant factors, along with several symptom-related PANSS items and neuropsychological measures.

Interestingly, despite the forensic group having lower cognitive scores and higher levels of personality comorbidity, they had better social functioning and fewer unmet needs according to the WHODAS and CAN/CANFOR assessments. These findings may reflect structural and institutional differences in the care provision between forensic and general psychiatric settings, where longer lengths of stay and higher staff-to-patient ratios in secure units may lead to greater levels of support and improved functional outcomes for the forensic group.

It must however be noted that the large overlap between comorbid personality disorder, particularly antisocial personality disorder and the forensic status limits the ability to attribute observed differences to forensic status per se rather than to underlying personality disorders. This point is particularly relevant in light of the documented importance of narcissistic dimensions of suicidality both in schizophrenia spectrum disorders and in antisocial and forensic populations.^28^ Notwithstanding this observation, collectively, our results indicate that suicide prevention efforts in patients with SSD should be sensitive to the different profiles of forensic and non-forensic populations. For forensic patients, targeted interventions should focus on personality pathology and enhancing functional autonomy, particularly in relational and social domains. In contrast, in non-forensic patients, trauma-informed care and integrated treatment for substance use disorders may be particularly impactful. Furthermore, our findings emphasise the importance of nuanced risk assessment that accounts for both shared and subgroup-specific predictors. While instruments like the OxMIS are useful when assessing suicide risk in individuals with SSD and bipolar disorder type I,^15 16^ forensic-specific variables not examined in our study, such as institutional support structures or legal uncertainty, warrant further exploration in future research.

In line with these considerations, the National Institute for Health and Care Excellence (NICE) in the UK has emphasised the importance of tailored, multi-agency interventions for suicide prevention in both community and custodial settings, particularly among those with a severe mental disorder, such as SSD, including forensic populations.^29^ Our findings, although mostly hypothesis-generating given the exploratory nature of the study, align with this perspective, underscoring the need for dynamic, functionally informed models of suicide prevention that are responsive to the distinct needs of both forensic and non-forensic patients with SSD.

### Limitations

Several limitations should be acknowledged: (1) the cross-sectional nature of our data limits causal inference and precludes conclusions about temporal relationships between different factors and self-harm; (2) the reliance on lifetime retrospective self-report and clinical records may introduce recall or reporting bias; (3) the use of only one simple binary item to assess self-harm and suicidal behaviour does not allow us to discriminate between these two concepts, nor to capture frequency, recency or severity of self-harm; (4) while the multinational design enhances generalisability, local differences in legal systems and forensic service structures may have introduced unmeasured heterogeneity; (5) because diagnoses were based on medical records rather than on structured interviews administered, it cannot be ruled out that psychiatric comorbidity has been underdiagnosed, particularly for axis-II disorders in the non-forensic population; (6) despite the robust use of LASSO regression to control for overfitting, the exploratory nature of the model calls for replication in independent samples; (7) like other penalised regression methods, the set of selected predictors may change due to sampling variability; finally, the statistical model used does not account for variability between countries in the refusal rate to participate in the study; (8) finally, forensic system-related variables, such as legal status, offence characteristics and legal uncertainty, were not available in our dataset, and warrant further investigation in future studies.

## Clinical implications

Our findings point to tailored approaches to risk assessment and intervention, with a particular focus on personality pathology, functional capacity in forensic settings, trauma exposure and clinical complexity in non-forensic populations. Given the cross-sectional design and exploratory modelling approach, these findings may provide a guide for future longitudinal studies aiming to clarify the causal pathways underlying these associations and to develop context-specific interventions aimed at reducing the high burden of suicidal behaviours in this vulnerable population.

## Data Availability

Data are available upon reasonable request. De-identified data will be shared on reasonable request directed to GdG (gdegirolamo@fatebenefratelli.eu) from qualified investigators with a signed data access agreement beginning 6 months and ending 5 years after publication.
