# Supplementary material for: Differentiating indicators of vulnerability to deliberate self-harm in forensic and non-forensic patients with schizophrenia spectrum disorders: a multinational LASSO-based analysis
Source: BMJ Ment Health. 2026 Sep 8;29(1):e302372. doi: 10.1136/bmjment-2025-302372 (PMC13584014; doi:10.1136/bmjment-2025-302372)
Supplement: Supplementary file 1 [file bmjment-29-1-s001.pdf]

Supplementary materials

TABLE 1S

LIST OF FORENSIC FACILITIES RECRUITING FOR THE EU-VIORMED PROJECT

COUNTRY	FACILITY NAME AND LOCATION	NUMBER OF FORENSIC BEDS	OFFICIAL SECURITY LEVEL
AUSTRIA	Justizanstalt Goellersdorf, Lower Austria, Goellersdorf	145	NA
	Klinik für Psychiatrie mit forensischem Schwerpunkt, Upper Austria, Linz	53	NA
GERMANY	Klinik für Forensische Psychiatrie und Psychotherapie, Zentrum für Psychiatrie Nordbaden, Wiesloch	258	NA
	Klinik für Forensische Psychiatrie des Pfalzklinikums, Klingenmünster	185	NA
	Klinik für Forensische Psychiatrie und Psychotherapie, Weinsberg	124	NA
ITALY	REMS, Centro Polifunzionale ex Ospedale Stellini, ULSS 9 'Scaligera', Nogara	130	NA
	Sistema Polimodulare REMS, Castiglione delle Stiviere	20	NA
	REMS Minerva, ASL Roma 5, Rome	20	NA
	REMS-D, Azienda USL Toscana Nord-Ovest, Volterra	20	NA
POLAND	Institute of Psychiatry and Neurology, Department of Forensic Psychiatry, Warsaw	60	Medium
	SP ZOZ Psychiatryc Hospital, Department of Forensic Psychiatry, Toszek,	40	Medium
	Psychiatric Hospital, Forensic Department, Starogard Gdański	40	Low
UNITED KINGDOM	South London and Maudsley NHS Foundation Trust, London	130	Low and Medium
	South West London and St. Georges Mental Health NHS Trust, London	75	Medium
	St. Andrew's Healthcare	240	Low and Medium

NA : Not Applicable, because there are no different security levels.
