# Supplementary material for: Differentiating indicators of vulnerability to deliberate self-harm in forensic and non-forensic patients with schizophrenia spectrum disorders: a multinational LASSO-based analysis
Source: BMJ Ment Health. 2026 Sep 8;29(1):e302372. doi: 10.1136/bmjment-2025-302372 (PMC13584014; doi:10.1136/bmjment-2025-302372)
Supplement: Supplementary file 2 [file bmjment-29-1-s002.pdf]

EU-VIORMED Clinical Studies
Patient Information Form_v.1.0_31_07_18

Patient ID: ______________________________________

euviormed PROJECT

Patient Information Form - PIF

To be completed for both cases and controls

Version 31.07.18

Examiner: ______________________________________

PIF_a1_Date form completed (dd/mm/yyyy): _________________

PIF_a2_Source(s) of information: please cross the appropriate box:

☐

code:	**0**	**1**	**2**	**3**	**4**	**5**
**PIF_Data source:**	Clinical charts/ clinicians	Family members	Patient	Both the patient and clinicians	Both the patient and family members	All – patient, clinicians and family members

PIF_01 *Type of subject* ☐

Control ……………………………………………………………………………………………..code=0

Case …………………………………………………………………………………….......... code=1

PIF_02 *Gender* ☐

Male…………………………………………………………………………………………………code=0

Female………………………………………………………………………………………………code=1

PIF_03 *Patient's current age* ☐☐

PIF_04 *Does the patient have any brothers or sisters?* ☐

No……………………………………………………………………………………………………code=0

Yes…………………………………………………………………………………………………...code=1

PIF_05 *Does the patient have any children?* ☐

No …………………………………………………………………………………………………code=0

EU-VIORMED Clinical Studies
Patient Information Form_v.1.0_31_07_18

Patient ID: ______________________________________

Yes ..……………………………………………………………………………………………….code=1

PIF_06 *Date of the most recent SSDs diagnosis (yyyy)* [ ][ ][ ][ ]

PIF_07 *Disease duration (years)* [ ][ ]

PIF_08 *Age at first contact with mental health services* [ ][ ]

PIF_09 *Type of SSDs as reported in current clinical charts (following DSM-5 criteria)* [ ]

Schizophrenia……..………………………………………………………………………………code=0
Schizoaffective disorders…………………………………………………………………...code=1
Delusional disorder ……………………………………………………………..........................code=2
Brief psychotic disorder………………………………………………………………………….code=3
Schizophreniform disorder……………………………………………………………………code=4
Drug-induced psychosis…………………………………………………………………………….code=5

PIF_10 *Co-morbidity with personality disorder as reported in clinical charts (following DSM-5 criteria)* [ ]

No……………………………………………………………………………………………………code=0
Yes ..……………………………..…………………………………………………………….code=1
Not known………………………………………………………………………………………..code=MISS

PIF_10a *If yes at PIF_10, type of co-morbid personality disorder (DSM-5 criteria)* [ ]

Paranoid…………….. ……………………......code=0
Schizoid……………………………………………………………………………………………code=1
Schizotypal…………………………………………………………………………………..code=2
Borderline……………………………………………………………………………………....code=3
Narcissistic………………………………………………………………………………..….code=4
Histrionic……………………………………………………………………………………….code=5
Antisocial……………………..code=6
Avoidant………………………………………………………………………………..……code=7
Dependent…………………………………………………………………………….......….code=8
Obsessive-Compulsive…………………………………………………………………..…….code=9
Not known……………………………………………………………………..………….code=MISS

Iozzino L, *et al. BMJ Ment Health* 2026; 29:e302372. doi: 10.1136/bmjment-2025-302372

EU-VIORMED Clinical Studies
Patient Information Form_v.1.0_31_07_18

Patient ID: ____

PIF_11 *Highest level of education attained*

No formal education……………………………………………………………………………..code=0

Some/completed primary school……………………………………………………………code=1

Some/completed secondary school…………………………………………………………code=2

Higher education (college, post-grad/professional)………………………………………code=3

PIF_12 *Years of full time education*

PIF_13 *Current marital status*

Married…………..…………………………………………………………………………….code=0

Single…………………………………………………………………………………………..code=1

Widowed…………………………………………………………………….………..…….code=2

Divorced……………………………………………………………………………….…. .code=3

PIF_14 *Highest occupational status ever achieved*

Never worked (including homemakers and students)……………………………………..code=0

Unskilled worker………………………………………………………………. ……………code=1

Skilled worker……………………………………………………………………………...…code=2

Professional…………………………………………………………………………….. …code=3

PIF_15 *Current social support, as people willing to help the patient if he/she needs help*

*(**<u>up to 2 main options allowed</u>**)*

None.……………………………………………………………………………………………..code=0

Family ……………………………..……………………………………………………….…code=1

Friends ………………………………………………………………………………………….code=2

Other patients ………..……………………………………………………………..……..…code=3

All……………..…………………………………………………………………………..… code=4

Not known………………………………………………………………………………...…code=MISS

PIF_16 *Does the patient have frequent personal contacts (at least twice a month) with relatives and/or friends who do not live with him/her?*

No…………………………………………………………………………………………..…..code=0

Yes …………………..……………………………….……………………………………..…..code=1

Not known……………………………………………………………………………………….code=MISS

Iozzino L, *et al. BMJ Ment Health* 2026; 29:e302372. doi: 10.1136/bmjment-2025-302372

EU-VIORMED Clinical Studies
Patient Information Form_v.1.0_31_07_18

Patient ID: ____

PIF_17 *Time spent doing nothing (lying on a sofa, watching TV, smoking, etc), not engaged in any structured activity*

Less than 3 hours a day ……………………………………………………………………………..code=0
Up to six hours a day……………………………………………………………………………code=1
More than 6 hours a day………………………………………………………………………..code=2
Not known………………………………………………………………………………………….code=MISS

PIF_18 *On regular psychotropic medications*

No……………………………………………………………………………………………...code=0
Yes…………………………………………………………………………………………...code=1

PIF_18a *If YES at PIF_18, second generation antipsychotics*

No………………………………………………………………………………………………. code=0
Yes……………………………………………………………………………………………… code=1

PIF_18b *If YES at PIF_18, clozapine*

No………………………………………………………………………………………………. code=0
Yes……………………………………………………………………………………………… code=1

PIF_18c *If YES at PIF_18, depot antipsychotics*

No………………………………………………………………………………………………. code=0
Yes……………………………………………………………………………………………… code=1

PIF_18d *If YES at PIF_18, first generation antipsychotics*

No………………………………………………………………………………………………. code=0
Yes……………………………………………………………………………………………… code=1

PIF_18e *If YES at PIF_18, antidepressants*

No……………………………………………………………………………………………….code=0
Yes…………………………………………………………………………………………… …code=1

PIF_18f *If YES at PIF_18, mood stabilizers (includes lithium, valproate, carbamazepine, lamotrigine, etc)*

No………………………………………………………………………………………………. code=0
Yes……………………………………………………………………………………………….code=1

Iozzino L, *et al. BMJ Ment Health* 2026; 29:e302372. doi: 10.1136/bmjment-2025-302372

EU-VIORMED Clinical Studies
Patient Information Form_v.1.0_31_07_18

Patient ID: ____

PIF_18g *If YES at PIF_18, benzodiazepines*

No……………………………………………………………………………………………….code=0

Yes…………………………………………………………………………………….. ……. code=1

PIF_19 *Living status (for controls)*

Home ……………………………………………………………………………………………code=0

Community-based residential facility ………………………………………………………..…….code=1

PIF_19a *If community-based residential facility at PIF_19, is there 24h staff cover*

No……………………………………………………………………………………………..code=0

Yes…………………………………………………………………………………………….code=1

PIF_19b *If community-based residential facilities at PIF_19, number of beds per room*

PIF_20 *Witnessed physical or sexual violence in his/her family*

No……………………………………………………………………………………………..code=0

Yes…………………………………………………………………………………………….code=1

Not known…………………….………………………………………………………………..code=MISS

PIF_20a *If yes at PIF_20: When did it occur?*

During early childhood (0-6 years)……………………………………………………………code=0

Childhood (7-12 years)………………………………………………………………………code=1

Adolescence (13-18 years)…………………………………………………………………...code=2

Adulthood………………………………………………………………………………………code=3

Not known…………………….…………………………………………………………………code=MISS

PIF_20b *If yes at PIF_20: How frequently*

Rarely…………………………………………………………………………………………code=0

Occasionally…………………………………………………………………………………code=1

Often……………………………………………………………………………………………code=2

Not known…………………….…………………………………………………………………code=MISS

PIF_20c *If yes at PIF_20: did someone in his/her family get medical attention because of this violence?*

No………………………………………………………………………………………………code=0

Yes……………………………………………………………………………………………….code=1

Iozzino L, *et al. BMJ Ment Health* 2026; 29:e302372. doi: 10.1136/bmjment-2025-302372

EU-VIORMED Clinical Studies
Patient Information Form_v.1.0_31_07_18

Patient ID: ______________________________

Not known……………………..……………………………………………………………………….code=MISS

PIF_21 *Victim of physical or sexual violence in his/her family*

No……………………………………………………………………………………………………….code=0

Yes……………………………………………………………………………………………………….code=1

Not known…………………………………..…………………………………………………………code=MISS

PIF_21a *If yes at PIF_21: When did it occur?*

During early childhood (0-6 years)…………………………………………………………………...code=0

Childhood (7-12 years)……………………………………………………………………………code=1

Adolescence (13-18 years)………………………………………………………………………….code=2

Adulthood………………………………………………………………………………………… code=3

Not known……………………..……………………………………………………………………code=MISS

PIF_21b *If yes at PIF_21: How frequently?*

Rarely……………………………………………………………………………………………………code=0

Occasionally…………………………………………………………………………………………….code=1

Often……………………………………………………………………………………………………..code=2

Not known……………………..…………………………………………………………………….code=MISS

PIF_21c *If yes at PIF_21: did the patient get medical attention because of this violence?*

No…………………………………………………………………………………………………….code=0

Yes……………………………………………………………………………………………………….code=1

Not known……………………..………………………………………………………………………code=MISS

PIF_22 *Was he/her ever been beaten, kicked or punched by someone?*

No……………………………………………………………………………………………………….code=0

Yes……………………………………………………………………………………………………….code=1

Not known………………………………………………………………………………………..……code=MISS

PIF_22a *If yes at PIF_22: When did it occur?*

During early childhood (0-6 years)…………………………………………………………………….code=0

Childhood (7-12 years)……………………………………………………………………………..code=1

Adolescence (13-18 years)…………………………………………………………………………….code=2

Adulthood………………………………………………………………………………………………code=3

Iozzino L, *et al. BMJ Ment Health* 2026; 29:e302372. doi: 10.1136/bmjment-2025-302372

Patient ID: ____

Not known………………………..……………………………………………………………….…code=MISS

PIF_22b *If yes at PIF-22: How frequently?*

Rarely………………………………………………………………………………………………code=0

Occasionally…………………………………………………………………………………….code=1

Often………………………………………………………………………………………………..code=2

Not known………………………..…………………………………………………………………code=MISS

PIF_22c *If yes at PIF_22; did the patient get medical attention because of this violence?*

No…………………………………………………………………………………………………….code=0

Yes……………………………………………………………………………………………………code=1

Not known………………………..………………………………………………………………….code=MISS

PIF_23 *Lifetime ever attempted suicide(s) or self-harm*

No…………………………………………………………………………………………..…….code=0

Yes……………………………………………………………………………………………..…code=1

Not known………………………..……………………………………………………………..……code=MISS

PIF_24 *Lifetime substance and alcohol use (**<u>up to 2 main options allowed)</u>***

Never……..………………………………………………………………………………………... code=0

Yes, reported by the patient……………………………………………….………………………...code=1

Yes, reported by family members…………………………………………………………………..code=2

Yes, diagnosis of abuse reported in clinical reports……………………………………………………code=3

Yes, diagnosis of dependence reported in clinical reports…………..……………………………….. code=4

Not reported in clinical records but reported by staff/informants……………………………………..code=5

Not reported, but positive from laboratory substance ……………………………………… ……….code=6

Not known………………………..………………………………………………………………code=MISS

PIF_25 *Current substance and/or alcohol use (past 12 months)*

No……………………………………………………………………………………………..…..code=0

Yes………………………. ……………………………..…………………………..………..…….code=1

Not known………………………..…………………………………………………………………code=MISS

PIF_26 *History of conviction or arrest for the index violence: institution of FIRST admission after the index violence (**for cases only**)*

Patient ID: ____

No……………………………………………………………………………………………………code=0

Yes (current forensic unit) ……………………………………………………………….............code=1

Yes (**another** forensic unit)……………………………………………………………..............code=2

Yes (prison)……..…………………………………………………………………….……………code=3

PIF_27 *History of any other violent behaviour lifetime (if Cases, in addition to the index offence)* ☐

No………………………………………………………………………………………………........code=0

Yes……………………………………………………………………………………………..code=1

Not known………………………………………………………………………………………code=MISS

PIF_27a *If YES at PIF_27, specify the offence type (**up to 3 options allowed**)* ☐

Robbery……………………………………………………………………………….................code=0

Kidnapping..……………………………………………………………………………………code=1

Stalking……………………………………………………………………………………..code=2

Assault with injury……………………………………………………………………………..code=3

Assault without injury………………………………………………………………….................code=4

Verbal violence – threat………………………………………………………………………..code=5

Other………………………………………………………………………………………..code=6

Not known………………………………………………………………………… ………code=MISS

PIF_27b *If YES at PIF_27, offence was made under the influence of alcohol or substances?* ☐

No……………………………………………………………………………….....................code=0

Yes…………………………………………………………………………………………...code=1

Not known…………………………………………………………………………………….code=MISS

PIF_27c *If YES at PIF_27, history of conviction or arrest* ☐

No…………………………………………………………………………………….…..........code=0

Yes……………………………………………………………………………………………..code=1

Not known………………………………………………………………………………………code=MISS

PIF_28 *Age at first admission in the lifetime in a forensic unit **(for controls NA)***

Not known………………………………………………………………………………………code=MISS

PIF_29 *Number of lifetime admissions in forensic units (1 or more; **for controls NA)*** ☐☐

Not known………………………………………………………………………………………code=MISS

Patient ID: ______________________________

PIF_30 *Total Time spent in forensic units in the lifetime (years;* ***for controls NA)***

Not known……………………………………………………………………………………………code=MISS

PIF_31 *Total Time spent in prison in the lifetime (years)*

Not known……………………………………………………………………………………….…code=MISS

PIF_32 *Collaboration skills in the last year*

Actively seeks treatment, willing to collaborate………………………………….. ………………….code=0

Wants to be helped, but lacks motivation……………………………………………………….........code=1

Passively accepts the treatment/intervention………………………………….……………..................code=2

Does not show attention or comprehension for the treatment efforts ………………………………..code=3

Actively refuses the treatment/intervention……………………………………………. ……………...code=4

Not known……………………………………………………………………………………..…code=MISS

PIF_33 *Did the patient withdraw from the study?*

No……………………………………………………………………………………………........code=0

Yes (Withdrawal Form to be completed)…………..…..……………………………………….............code=1

Iozzino L, *et al. BMJ Ment Health* 2026; 29:e302372. doi: 10.1136/bmjment-2025-302372
