# Supplementary material for: Differentiating indicators of vulnerability to deliberate self-harm in forensic and non-forensic patients with schizophrenia spectrum disorders: a multinational LASSO-based analysis
Source: BMJ Ment Health. 2026 Sep 8;29(1):e302372. doi: 10.1136/bmjment-2025-302372 (PMC13584014; doi:10.1136/bmjment-2025-302372)

S3. ***Sensitivity analysis excluding CAN-FOR item 10***

Because CAN-FOR item 10 assesses self-harm behaviours and suicidal ideation, it may partially overlap with the study's outcome of lifetime suicide attempts. To determine whether including this item influenced the identification of predictors in the LASSO models, a sensitivity analysis was conducted by excluding this variable from the predictor set. CAN-FOR item 10 is scored on a three-level scale (0–2), with higher scores indicating a greater clinical need related to self-harm behaviours or suicidal ideation. In the present sample, the highest response category (score = 2), which indicates the presence of suicidal ideation and/or self-harm behaviour, was reported by 8.2% of forensic participants and 3.5% of non-forensic participants. The LASSO analyses were then repeated separately for forensic (Figure S3) and non-forensic subsamples (Figure S4) after removing CAN-FOR item 10 from the design matrix. The resulting models demonstrated a similar pattern of selected predictors as observed in the primary analyses. Only minor differences were noted in the ranking or inclusion of a small number of variables, while the main predictors identified in the primary models remained unchanged. These findings suggest that the predictors identified in the main analyses were not influenced by the inclusion of CAN-FOR item 10, indicating that the results are robust even without this potentially overlapping variable.

FIGURE 3S

LASSO REGRESSION ANALYSIS OF FACTORS ASSOCIATED WITH SELF-HARM BEHAVIORS IN FORENSIC PATIENTS EXCLUDING CAN/CANFOR ITEM 10 (N = 220)

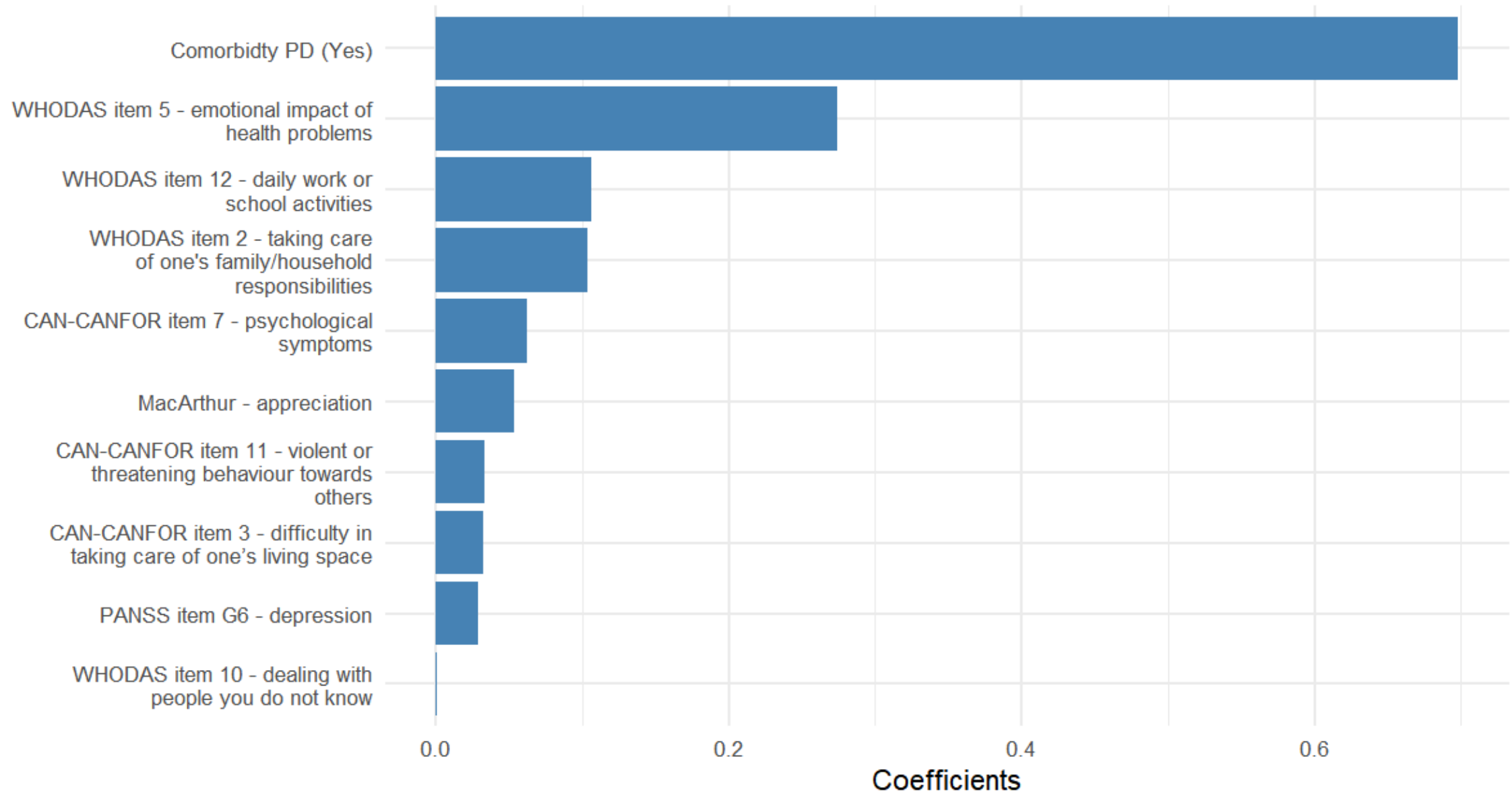

FIGURE 4S

LASSO REGRESSION ANALYSIS OF FACTORS ASSOCIATED WITH SELF-HARM BEHAVIORS IN NON-FORENSIC PATIENTS EXCLUDING CAN/CANFOR ITEM 10 (N = 172)

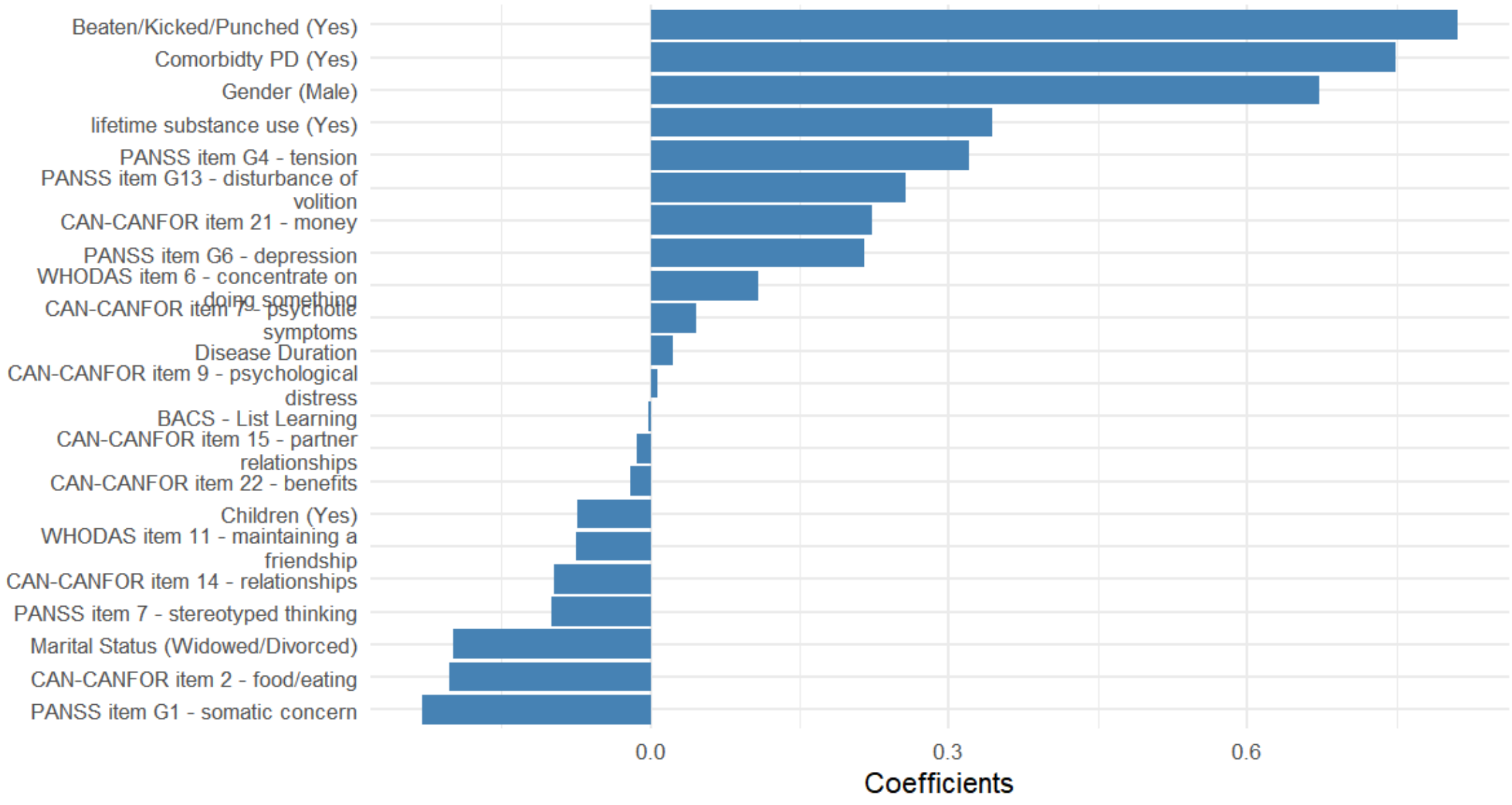

Supplement: Supplementary file 3 [file bmjment-29-1-s003.pdf]
